# Presentation, management, and outcomes of sepsis in adults and children admitted to a rural Ugandan hospital: A prospective observational cohort study

**DOI:** 10.1371/journal.pone.0171422

**Published:** 2017-02-15

**Authors:** Kristina E. Rudd, Leonard K. Tutaryebwa, T. Eoin West

**Affiliations:** 1 International Respiratory and Severe Illness Center, University of Washington, Seattle, Washington, United States of America; 2 Division of Pulmonary and Critical Care Medicine, Department of Medicine, University of Washington, Seattle, Washington, United States of America; 3 Department of Paediatrics and Child Health, Bwindi Community Hospital, Kanungu, Uganda; Tulane University, UNITED STATES

## Abstract

**Objectives:**

Limited data are available on sepsis in low-resource settings, particularly outside of urban referral centers. We conducted a prospective observational single-center cohort study in May 2013 to assess the presentation, management and outcomes of adult and pediatric patients admitted with sepsis to a community hospital in rural Uganda.

**Methods:**

We consecutively screened all patients admitted to medical wards who met sepsis criteria. We evaluated eligible patients within 24 hours of presentation and 24–48 hours after admission, and followed them until hospital discharge. In addition to chart review, mental status evaluation, peripheral capillary oxygen saturation, and point-of-care venous whole blood lactate and glucose testing were performed.

**Results:**

Of 56 eligible patients, we analyzed data on 51 (20 adults and 31 children). Median age was 8 years (IQR 2–23 years). Sepsis accounted for a quarter of all adult and pediatric medical ward admissions during the study period. HIV prevalence among adults was 30%. On enrollment, over half of patients had elevated point-of-care whole blood lactate, few were hypoglycemic or had altered mental status, and one third were hypoxic. Over 80% of patients received at least one antibiotic, all severely hypoxic patients received supplemental oxygen, and half of patients with elevated lactate received fluid resuscitation. The most common causes of sepsis were malaria and pneumonia. In-hospital mortality was 3.9%.

**Conclusions:**

This study highlights the importance of sepsis among adult and pediatric patients admitted to a rural Ugandan hospital and underscores the need for continued research on sepsis in low resource settings.

## Introduction

Infectious diseases are among the leading causes of morbidity and mortality worldwide, with the greatest burden in low-income countries (LICs) [1][2]. In Uganda, a sub-Saharan African LIC of 37.8 million people, HIV/AIDS, pneumonia, diarrheal diseases, and malaria account for 4 of the top 5 causes of death [1][3]. Sepsis, the dysregulated host response to infection, is a common syndrome encountered by clinicians treating patients with infection. Despite what is known about the epidemiology of these infectious diseases at the population level, few data are available to inform the clinical management of sepsis in low-resource settings such as Uganda [4].

The Surviving Sepsis Campaign (SSC) has published guidelines for management of patients with severe sepsis and septic shock [5]. While these guidelines have become the standard of care in high-resource settings, much of the world remains unable to routinely implement them either in part or in full [6–10]. While efforts have been made to provide modified recommendations and approaches for limited-resource settings [2,11–18], a deeper understanding of the current state of care in more varied low-resource settings is paramount to allow clinicians and public health practitioners to improve care and better target research efforts.

While studies describing the management of sepsis in low-resource settings have been published previously, many of these have excluded pediatric patients, evaluated patients with acute febrile illness instead of sepsis specifically, focused on patients with just one infectious disease such as malaria or pneumonia, or limited their patient population to those in urban referral centers [19–27]. Beyond observational studies, others have published results of interventional trials for sepsis or shock conducted in sub-Saharan Africa [17,28,29]. Important work has been done in Uganda evaluating the presentation, management and outcomes of adult patients with severe sepsis presenting to urban referral hospitals [30–40]. Few studies have evaluated non-neonatal pediatric sepsis in Uganda or sepsis in rural or non-tertiary care hospitals in LICs. To address these knowledge gaps, we conducted a prospective observational single-center cohort study with the primary objective of describing the epidemiology, management, and outcomes of sepsis in adults and children presenting to a rural Ugandan hospital.

## Methods

### Study design

We conducted a prospective observational cohort study at Bwindi Community Hospital, a private not-for-profit 112-bed hospital serving a catchment area of approximately 100,000 patients in rural southwest Uganda. This is the only hospital in the area and serves a very poor and rural community. The hospital has both inpatient and outpatient facilities including well-developed HIV treatment and prevention services. Hospital capabilities include full obstetric services, surgical facilities with a general surgeon available 24 hrs per day, and dedicated adult and pediatric wards with medical officers on-site during the day and available by call overnight. There is a reliable supply of IV fluids and sterile supplies; basic antibiotics including penicillins, gentamycin, and ceftriaxone; IV dextrose; antimalarials and tuberculosis treatments; and supplemental oxygen in the form of 6L concentrators (2 per ward, sometimes requiring splitting of the flow between patients). The hospital has basic laboratory services, basic radiography services, and one of the only blood banks in the region. Malaria is diagnosed with rapid diagnostic testing and blood smear. While limited microbiology testing such as gram stain is available, microbiological culture and sensitivity testing is not available on-site, nor are there more advanced imaging technologies such as computed tomography (CT). The nearest referral hospital is approximately 180km away.

We consecutively enrolled all adult (age ≥ 18yrs) and pediatric (ages 28d – 17yrs) patients admitted to the inpatient medical wards who met sepsis criteria May 4–26, 2013. Sepsis was defined as the presence of the systemic inflammatory response syndrome (SIRS) and suspected infection. For adults, SIRS was defined as two of the four following criteria: temperature greater than 38°C (100.4°F) or less than 36°C (96.8°F), heart rate of more than 90 beats per minute, respiratory rate of more than 20 breaths per minute or arterial carbon dioxide tension (PaCO_2_) of less than 32mm Hg, and abnormal white blood cell count (>12,000/μL or < 4,000/μL or >10% immature [band] forms) [41]. For children, SIRS was defined as two of the four following criteria: temperature greater than or equal to 38.5°C or less than 36°C, heart rate > 2 standard deviations (SD) above normal for age in the absence of external stimulus, respiratory rate > 2 SD above normal for age, and white blood cell count elevated or depressed for age, or > 10% bands [42]. Although standard definitions for pediatric SIRS use core temperature, only axillary temperatures are obtained at the study hospital, and thus axillary temperature were substituted. Of note, to meet pediatric SIRS criteria, children must have a temperature or WBC abnormality in addition to at least one other SIRS criterion. Surgical patients, women admitted to the maternity ward, and neonates (less than 28 days of age) were excluded, as were patients who did not meet sepsis criteria, did not provide consent, or were not enrolled within 24 hours of admission.

Potential subjects were identified through chart review for all new admissions to the adult and pediatric wards at least twice daily. Qualifying patients and/or their legal representatives were then approached for consent and study enrollment. Data were collected through chart review as well as through direct patient assessment by study staff as soon as possible within 24 hours of presentation and again 24–48 hours after admission. Additional chart review was performed upon hospital discharge. Information obtained from chart review included demographic information; admission and discharge diagnoses; vital signs; laboratory findings (CBC with differential, basic chemistry, HIV status, CD4 count, malaria rapid test and smear, and gram stain); results of radiology studies; type and quantity of administered IV fluid or antimicrobials; administration of supplemental oxygen; complications; disposition; and length of stay. Additional data obtained through direct patient assessment included age-appropriate AVPU and Glasgow Coma scores (GCS), peripheral capillary oxygen saturation (SpO2), point-of-care lactate testing of whole venous blood (Lactate Plus lactate meter, Nova Biomedical), and point-of-care glucose testing of whole venous blood (ACCU-CHECK Aviva glucometer, Roche). All abnormal glucose, lactate, and SpO2 results identified were reported to the patient’s treating clinician within one hour of the test.

### Statistical analysis

Data was recorded manually in case report forms. We performed statistical analysis using Stata/IC 13. Continuous variables with normal distributions are expressed as means with standard deviations (SD), continuous variables with non-normal distributions are expressed as medians with interquartile range (IQR), and categorical variables are expressed as counts with percentages.

### Human subjects and ethics

Ethical approval was obtained from the Human Subjects Division of the University of Washington (Seattle, WA, USA) and the Mbarara University of Science and Technology Institutional Review Committee (Mbarara, Uganda). Additional study approval was obtained from the Ugandan National Council of Science and Technology (UNCST). Informed consent was obtained from all adult patients and a parent of all pediatric patients (less than 18 years of age) in Rukiga, the local language. In the event that an adult patient was unable to provide consent due to altered mental status, their legal representative provided informed consent. In the event that patients or their legal representatives were able to read, consent was obtained in writing. In the event that patients or their legal representatives were unable to read, the consent form was read aloud by an interpreter. Patients or representatives who were unable to sign their name drew an “X” on the consent form signature line or marked the space with a thumbprint and a witness signed the form.

## Results

**Fig 1:**
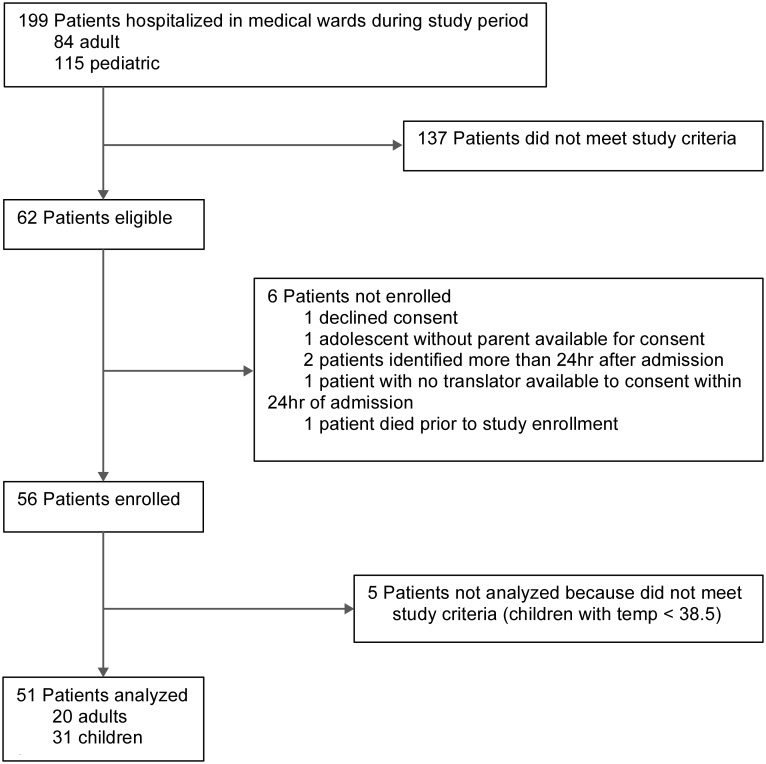
Patient identification.

During the study period, 84 adult and 115 pediatric patients were admitted to the adult and pediatric medical wards (Fig 1). Of these, 62 met enrollment criteria and 56 patients were enrolled in the study. One patient who met study criteria died prior to enrollment. Five patients were subsequently excluded from the analysis as re-review indicated that they did not meet inclusion criteria. Therefore, of 56 eligible patients we analyzed data for 51 patients (20 adults and 31 children), representing 23.8% (20/84) of all adult and 27% (31/115) of all pediatric patients admitted to the hospital during the study period.

The characteristics of subjects at enrollment are given in Table 1. The median age was 8 years (IQR 2–23 years). Among adults, the median age was 32 years (IQR 22–42 years). Among children, the median age was 3 years (IQR 2–7 years). There was a roughly equal distribution of males and females in both the adult and pediatric cohorts. All of the study subjects were of the Bantu ethnic majority. Only eight patients (16%) had a CBC drawn upon admission, and thus most adult and all pediatric patients met sepsis criteria for study enrollment exclusively based on vital sign abnormalities. No children had significant co-morbidities. Among adults, the most common co-morbidity was HIV (n = 6, 30.0%). The most common chief complaints among adults were fever (75.0%) and neurologic symptoms (65.0%). The most common chief complaints among children were fever (93.5%) and cardiopulmonary symptoms (71.0%).

**Table 1:** Characteristics of patients.

Variable	Adults	Pediatrics
**Number of patients, *n* (%)**	20 (39.2%)	31 (60.8%)
**Male gender, *n* (%)**	11 (55.0%)	14 (45.2%)
**Ethnicity, *n* (%)**		
Bantu	20 (100.0%)	31 (100.0%)
Batwa	0 (0.0%)	0 (0.0%)
**Median age, yr [IQR]**	32 [22–42]	3 [2–7]
**SIRS criteria, *n* (%)**		
Heart rate	18 (90.0%)	19 (61.3%)
Respiratory rate	13 (65.0%)	29 (93.5%)
Temperature	15 (75.0%)	31 (100.0%)
White blood cell count	2 (10.0%)	0 (0.0%)
**Co-morbidities, *n* (%)**		
Diabetes	1 (5.0%)	0 (0.0%)
HIV	6 (30.0%)	0 (0.0%)
Tuberculosis	1 (5.0%)	0 (0.0%)
**Chief complaint, *n* (%)**		
Anorexia	0 (0.0%)	10 (32.3%)
Cardiopulmonary^a^	9 (45.0%)	22 (71.0%)
Fever	15 (75.0%)	29 (93.5%)
Gastrointestinal^b^	9 (45.0%)	9 (29.0%)
Musculoskeletal^c^	3 (15.0%)	2 (6.5%)
Neurologic^d^	13 (65.0%)	10 (32.3%)
Upper respiratory symptoms^e^	0 (0.0%)	4 (12.9%)

^a^Chest pain, cough, dyspnea, hemoptysis.

^b^Abdominal pain, diarrhea, vomiting.

^c^Arthralgias, back pain, extremity pain, myalgias.

^d^Altered mental status, dizziness, headache, seizure.

^e^Coryza, rhinorrhea.

The characteristics of subjects at the time of enrollment (within 24 hours of admission) are given in Table 2. One adult (5.0%) and two children (6.5%) were hypoglycemic. The majority of subjects had evidence of hypoperfusion, especially adults, eight (40%) of whom had a lactate > 4 mmol/L. Of those adults with initial blood pressure recorded, six (30%) were hypotensive (SBP < 90 mmHg), two of whom had a lactate > 4 mmol/L and three of whom had a lactate > 2 and ≤ 4 mmol/L. Blood pressure was not recorded in children. Nine adults (45%) and 11 children (35.5%) were hypoxemic (SpO2 < 94%). The majority of patients had normal mental status upon assessment but altered mental status was more common in children.

**Table 2:** Severity of illness at 24 and 48 hours.

Variable	Adults		Pediatrics	
	24 hrs N = 20	48 hrs N = 16	24 hrs N = 31	48 hrs N = 30
**Hypoglycemia, BG < 70 mg/dL, *n* (%)**^a^	1 (5.0%)	0 (0.0%)	2 (6.5%)	3 (10%)
**Hypoperfusion, *n* (%)**^b^				
Mild, lactate > 2 and ≤ 4 mmol/L	6 (30.0%)	8 (50.0%)	14 (45.2%)	13 (43.3%)
Severe, lactate > 4 mmol/L	8 (40.0%)	3 (18.8%)	5 (16.1%)	2 (6.7%)
**Hypotension, SBP < 90mmHg, *n* (%)**^c^	6 (30.0%)	4 (25.0%)	-	-
**Hypoxia, SpO2 < 94%, *n* (%)**^d^	9 (45.0%)	1 (6.3%)	11 (35.5%)	5 (16.7%)
**Mental status**				
AVPU score, *n* (%)				
A	16 (80.0%)	15 (93.8%)	20 (64.5%)	28 (93.3%)
V	2 (10.0%)	0 (0.0%)	5 (16.1%)	0 (0.0%)
P	1 (5.0%)	0 (0.0%)	5 (16.1%)	1 (3.3%)
U	1 (5.0%)	1 (6.3%)	1 (3.2%)	1 (3.3%)
**Glasgow Coma Score, median [IQR]**	15 [15–15]	15 [15–15]	15 [11–15]	15 [15–15]

^a^Missing value for 1 adult at 24 hrs.

^b^Missing values for 1 adult at 24 hrs and 1 adult at 48 hrs.

^c^Pediatric blood pressure not available. Missing values for 6 adults at 24 hrs and 2 adults at 48 hrs.

^d^Due to lack of access to infant-specific oximetry probes, missing values for 6 children at 24 hrs and 7 children at 48 hrs.

On follow-up assessment (24–48 hours after admission), four adults (20%) and one (3.2%) pediatric patient had been discharged home in improved condition; none had died or been transferred. Of those patients still hospitalized, no adults and three children (10%) were hypoglycemic. Compared to enrollment, fewer patients had severe hypoperfusion but eleven adults (68.8%) and 15 children (50.0%) had persistently elevated lactates. A quarter of adults remained hypotensive. Prevalence of hypoxia decreased in both adults and children. Nearly all subjects had a normal mental status.

The majority of adult (80%) and pediatric (83.9%) patients received at least one antibiotic during their admission. Antibiotics most commonly prescribed were ceftriaxone in adults (45%) and chloramphenicol (38.7%), ceftriaxone (29%), gentamicin (29%) and ampicillin (25.8%) in children (Table 3). Eight adults (40%) and eight children (25%) received an antimalarial agent. All adult and pediatric patients who were severely hypoxic (SpO2 < 90%) received supplemental oxygen support. Fluid resuscitation was defined as ≥2 L of isotonic intravenous fluid in adults or ≥ 20 mL/kg in children within the first 24hr of admission. At least half of adults and children with evidence of severe hypoperfusion received fluid resuscitation.

**Table 3:** Patient management.

Variable		Adults N = 20	Pediatrics N = 31
**Antimicrobials received during admission**			
	**Antibiotic, *n* (%)**	16 (80.0%)	26 (83.9%)
	Amoxicillin	1 (5.0%)	1 (3.2%)
	Ampicillin	0 (0.0%)	8 (25.8%)
	Ceftriaxone	9 (45.0%)	9 (29.0%)
	Chloramphenicol	3 (15.0%)	12 (38.7%)
	Ciprofloxacin	1 (5.0%)	0 (0.0%)
	Dicloxacillin	1 (5.0%)	0 (0.0%)
	Doxycycline	1 (5.0%)	0 (0.0%)
	Erythromycin	0 (0.0%)	1 (3.2%)
	Gentamicin	2 (10.0%)	9 (29.0%)
	Penicillin G	0 (0.0%)	1 (3.2%)
	Trimethoprim-Sulfamethoxazole	3 (15.0%)	0 (0.0%)
	**Antihelminth, *n* (%)**	1 (5.0%)	2 (6.5%)
	Albendazole	1 (5.0%)	1 (3.2%)
	Mebendazole	0 (0.0%)	1 (3.2%)
	**Antimalarial, *n* (%)**	8 (40.0%)	8 (25.0%)
	Artemether	5 (25.0%)	8 (25.8%)
	Quinine	5 (25.0%)	0 (0.0%)
	**Antiviral, *n* (%)**	0 (0.0%)	0 (0.0%)
**Oxygen support if hypoxic, *n* (%)**^a^			
	SpO2 < 94%	4/9 (44.4%)	9/11 (81.2%)
	SpO2 < 90%	3/3 (100.0%)	5/5 (100%)
**IV fluid bolus if hypoperfused, *n* (%)**^b^			
	Mild, venous lactate > 2 and ≤4 mmol/L	3/7 (42.9%)	8/14 (57.1%)
	Severe, venous lactate > 4 mmol/L	4/8 (50.0%)	3/5 (60.0%)

^a^In first 24hr of admission.

^b^Mild hypoperfusion defined as systolic blood pressure < 90mmHg (in adults) or venous lactate > 2 and ≤ 4 mmol/L in the first 24hr of admission. Severe hypoperfusion defined as systolic blood pressure < 70 mmHg (in adults) or venous lactate > 4 mmol/L in the first 24hr of admission. Fluid bolus defined as ≥ 2L isotonic crystalloid in first 24hr in adults and ≥ 20 mL/kg isotonic crystalloid in the first 24hr in pediatrics.

In adults the most common causes of sepsis, as documented in the medical record, were malaria (35%) and pneumonia (20%) (Table 4). The most common causes of sepsis in children were pneumonia (48.4%) and malaria (29%). Seven children (22.6%) were diagnosed with viral upper respiratory tract infections (URTI); two of these had co-infection with malaria or bacterial infections. Four of five (80%) of the children with viral URTI alone had evidence of hypoperfusion (lactate > 2 mmol/L) at enrollment. Two adult (10%) and no pediatric patients died in hospital or were discharged home in moribund condition. No adults and one child (3.2%) were transferred to other medical facilities. The patient who was transferred was in serious condition and was transferred to the nearest referral hospital with CT capabilities, approximately 180km away. The median hospital length of stay was 2.5 days for adults (IQR 2.0–3.5) and 2 days for children (IQR 2–4).

**Table 4:** Patient outcomes.

Variable	Adults N = 20	Pediatrics N = 31
**Discharge diagnosis, *n* (%)**		
Acute otitis media	0 (0.0%)	2 (6.5%)
Brucellosis	0 (0.0%)	1 (3.2%)
Encephalitis	0 (0.0%)	2 (6.5%)
Gastroenteritis	1 (5.0%)	1 (3.2%)
Idiopathic abdominal pain^a^	1 (5.0%)	0 (0.0%)
Malaria	7 (35.0%)	9 (29.0%)
Pharyngotonsillitis	0 (0.0%)	1 (3.2%)
Pneumonia	4 (20.0%)	15 (48.4%)
Prostatitis	1 (5.0%)	0 (0.0%)
Puerperal sepsis	1 (5.0%)	0 (0.0%)
Sepsis with unspecified source	3 (15.0%)	0 (0.0%)
Toxoplasmosis	1 (5.0%)	0 (0.0%)
Tuberculosis	1 (5.0%)	0 (0.0%)
Typhoid fever	1 (5.0%)	1 (3.2%)
Viral upper respiratory tract infection	1 (5.0%)	7 (22.6%)
**Length of stay, median days [IQR]**	2.5 [2.0–3.5]	2.0 [2.0–4.0]
**Disposition, *n* (%)**		
Home in improved condition	17 (85.0%)	30 (96.8%)
Home to die	1 (5.0%)	0 (0.0%)
Died in hospital	1 (5.0%)	0 (0.0%)
Transferred to other facility	0 (0.0%)	1 (3.2%)
Left against advice	1 (5.0%)	0 (0.0%)

^a^This patient left against medical advice prior to complete evaluation.

## Discussion

This study demonstrates that sepsis is a common reason for hospital admission in rural Uganda, accounting for about a quarter of all medical admissions. Adult patients are young and HIV is a common co-morbidity. In both adults and children, sepsis is frequently attributed to malaria and pneumonia. Despite high rates of hypoxemia and hypoperfusion on presentation, hospital mortality is low.

This study adds important knowledge to the fields of adult and pediatric tropical medicine and global health. Many previous studies of sepsis in sub-Saharan Africa have been conducted with urban patient populations, usually at major regional or national hospitals, and have frequently excluded children. Our results differ from prior studies in a number of ways. First, subjects in our study were relatively well, with a lower prevalence of HIV and other chronic diseases relative to that reported elsewhere in the literature [21–23,26,30,31,34,43]. The inpatient HIV testing rate is > 85% in this hospital, making it unlikely that a significant number of study patients had undiagnosed HIV. The lower HIV prevalence in this cohort may be in part due to the hospital’s aggressive HIV testing and treatment strategy and the rural nature of the population. The overall HIV prevalence in the Southwestern Uganda region is 8.0% (as of 2011) [44], whereas the prevalence of HIV among 349 pediatric (under 16yr) and 558 adult inpatients tested through this hospital in 2013 was only 0.9% in children and 5.0% in adults [45]. Additionally, chronic comorbidities among study subjects are likely underestimates because most patients in this community do not get preventative care and conditions such as diabetes, tuberculosis and hypertension are frequently not diagnosed until complications develop. Our inclusion of patients with non-severe sepsis is an important contrast to other studies of sepsis in sub-Saharan Africa which restricted inclusion criteria to subjects with severe sepsis or shock [17,28].

Second, we found a higher prevalence of malaria among our cohort than others have reported elsewhere in Uganda [31,32,35,37]. There may be several reasons for this. All patients diagnosed with malaria underwent rapid testing and blood smear by treating providers to confirm parasitemia but this was not independently tested. Additionally, the more rural nature of the area compared to the sites of other studies may account for our higher malaria prevalence than previously reported [37].

Third, the low mortality rate that we identified is a significant contrast to the high case-fatality rates reported elsewhere [30–33,35], and is likely in part due to the relatively well patient population enrolled. However, it is unlikely that this entirely explains our findings, as a large number of patients had evidence of end-organ dysfunction upon initial evaluation. While Bwindi Community Hospital is the only hospital in this area, it is private and well-resourced relative to public hospitals of similar size in Uganda.

Most patients in this study received antimicrobials and, where indicated, supplemental oxygen. Fluid resuscitation was less frequent even in more severely ill patients: only 50% of adults and 60% of children with severely elevated venous lactate received IV fluid boluses. One unique feature of the study site is that nurses frequently initiate IV fluids and antibiotics for ill patients who present directly to the wards overnight.

Strengths of our study are that we consecutively screened for all patients with sepsis requiring medical admission during the study period. Enrollment of eligible patients was high. Full medical record review was completed on all patients and no patients were lost to follow up. There was minimal missing data, and is noted where applicable. Therefore, although our study is small, our results are likely to be robust. In addition, our study is one of only a few to specifically describe pediatric sepsis in sub-Saharan Africa.

This study has several limitations. The study was designed to be strictly observational, but the presence of medically-trained study staff and the collection of primary data likely affected patient management and outcomes. We collected—and reported to treating clinicians—clinical data which were not routinely measured in non-study patients (e.g. pulse oximetry, point of care glucose, and point of care venous lactate). In several cases, enrolled patients then received interventions such as supplemental oxygen, IV dextrose or IV fluid boluses based on study findings.

Another limitation of this study was our strict application of the definition of pediatric sepsis [42] to this limited-resource setting. At least five pediatric patients were initially enrolled but were not included in the analysis because their temperature was elevated but less than 38.5°C, the cutoff for SIRS criteria in children [42]. We maintained this standard temperature cutoff despite the site’s use of axillary rather than core temperature measurement for pediatric patients, and thus likely missed several patients with true sepsis physiology who may have had a core temperature that met inclusion requirements. Additionally, because the study site does not commonly perform CBCs on children (only 2 of 31 pediatric patients included in analysis had CBCs performed at all, neither of which met criteria for sepsis) and the pediatric sepsis definition requires either elevated temperature or WBC derangement, it’s likely that several other patients with sepsis physiology but no fever were excluded. Additionally, we excluded pregnant women, and did not screen postpartum women admitted to the Maternity Ward or septic patients requiring surgical intervention. We excluded neonates because neonatal sepsis is a separate syndrome with unique etiology, presentation, management guidelines, and outcomes that significantly differ from sepsis in older children. These limitations may have resulted in underestimates of the prevalence of sepsis and mortality rates from sepsis, and potentially widens an existing gap in research on these populations.

The small size of our study population and the low mortality rate precluded determination of correlates of death or of hospital length of stay. Additionally, likely due to the small size of this study, we failed to enroll any Batwa patients. This is a marginalized minority population, and the presentation and outcomes of sepsis has not previously been described in this community. Although the study hospital is a major source of medical care for the Batwa community in southwest Uganda, failure to enroll any of these individuals was probably due to the small study size.

Since this study was performed, an updated consensus definition for adult sepsis has been released [46]. Notably, the definition for pediatric sepsis used in this study is consistent with current definitions. Although our study used the adult sepsis definition that was current at the time the study was performed, we acknowledge that our use of a now-outdated definition limits the future comparability and generalizability of our study.

Lastly, while the external validity of this study is high in terms of presentation of sepsis among adult and pediatric patients in similar rural areas in Sub-Saharan African LICs, given that this was a private hospital which is relatively well resourced, the generalizability of sepsis management and outcomes may be limited.

This study is one of the first to evaluate the presentation, management and outcomes of sepsis among both adults and children in a rural community in Uganda. Our results highlight the burden of sepsis in this population. Furthermore, our findings emphasize the necessity of continued sepsis research in sub-Saharan Africa and other low resource settings around the world.

## Supporting information

S1 File Data. (XLS)
